# Characterization, Sensory and Oxidative Stability Analysis of Vegetable Mayonnaise Formulated with Olive Leaf Vinegar as an Active Ingredient

**DOI:** 10.3390/foods11244006

**Published:** 2022-12-11

**Authors:** Antonella De Leonardis, Vincenzo Macciola, Ayesha Iftikhar, Francesco Lopez

**Affiliations:** Department of Agricultural, Environmental and Food Sciences (DiAAA), University of Molise, Via De Sanctis, 86100 Campobasso, Italy

**Keywords:** mayonnaise, innovative vinegar, olive leaf, oxidative stability, sensory analysis

## Abstract

Development of novel food products represents a basic meeting point for health and business requirements. Mayonnaise sauce is well-suited to be a healthy and tasty dressing. In this study, mayonnaise was formulated by using unconventional ingredients, such as olive leaf vinegar (OLV), soybean/high oleic sunflower oil blend, and soymilk (as an egg substitute). An 18% alcoholic vinegar was used as the control sample. OLV is a rich source of bioactive substances, especially polyphenols and represents a possible way to enhance the olive oil by-product valorisation. For this new typology of vinegar an high level of phenolic compounds (7.2 mg/mL GAE), especially oleuropein (6.0 mg/mL oleuropein equivalent) was found. OLV mayonnaise had 57% fat, composed of 11%, 64%, and 23% saturated, monounsaturated, and polyunsaturated fatty acids, while linolenic acid was up to 1.7%. The phenol and oleuropein contents were 68 and 52 mg/100 g, respectively. Sensory panellists expressed a moderate overall acceptability for both samples but attested more distinctive and positive sensations for the colour, odour, and taste attributes of OLV mayonnaise. Finally, oxidative stability and shelf life were better in OLV mayonnaise than in the control. Specifically, the peroxide value remained low (around 4.5 meqO_2_/kg) after 12 months of storage at room and low (4 °C) temperatures.

## 1. Introduction

Mayonnaise is a very popular and widely consumed sauce as a seasoning and a condiment. Historically, a mayonnaise-like sauce was produced traditionally in the city of Mahon (Spain), from which its name is derived probably(Mahon-naise) [1]. Commercial production of mayonnaise originated in early 1900s, and it recently achieved a global market of about US$11.8 billion [2].

Conventionally, mayonnaise is prepared with oil, egg yolks, acidifying agent, salt, and various seasonings (optional). The colour of mayonnaise is usually pale yellow or sometimes it could be white, with consistency varying depending on the ingredients [3]. From the colloidal point of view, mayonnaise is an oil-in-water (O/W) low-pH emulsion with 65–80% oil content. Both the low pH and high fat content make mayonnaise a microbe-stable emulsion even at room temperature [4,5].

Generally, emulsifying agents are added to prevent the separation of phases; egg phospholipids perform this function in mayonnaise [6]. Emulsion systems are mostly used to deliver functional compounds and develop a novel functional food [7,8,9].

Even though the global market value of mayonnaise and similar sauces is continuously growing, health concerns about the composition of conventional mayonnaise are still increasing because of its high total calorie value, intake of cholesterol deriving from eggs, and rapid auto-oxidation of unsaturated fatty acids of the lipid fraction [10,11,12]. These issues are at odds with the expanding worldwide demand for natural, healthy, and more nutritional food products that today represents an essential point to meet health and business requirements. Therefore, the development of new suitable food products is rapidly increasing due to consumer requests for healthy diet food products that fits well the stringent need linked with the production of low-calorie food. Therefore, formulating an innovative and healthier mayonnaise, such as egg-less, vegan, low-fat, organic, and other variants, is a very current challenge [1,13]. Several studies have been conducted to accomplish this goal. The functionality of mayonnaise has been improved by the addition of mustard, a source of isothiocyanates that provide more stability during long-term storage [14]. Mayonnaise prepared with extra virgin olive oils exhibited better rheological characteristics, but was also inversely correlated with a very high phenolic content [15]. In similar studies, different concentrations of apple peel extract were introduced to mayonnaise to improve oxidative stability, sensory attributes, rheological characteristics, and whole quality [16].

In the current scenario, the demand for environmentally sustainable food is as necessary to address as that for healthy food. In this contest, valorisation of agrifood by-products represents an important practice for reducing generated wastes and implementing a circular economic model with increasing environmental protection. Among other uses, these by-products can be considered as an alternative source of natural additives or ingredients for food products. Olive leaves (*Olea europaea* L.) are an abundant promising by-product of the olive oil industry, generated both during tree pruning and olive fruit harvesting [17,18]. Traditionally, olive leaves have been used in popular medicine to prevent and treat several diseases especially those related to the kidney, heart and liver. Indeed, olive leaves contain high quantities of bioactive substances (listed below) that exhibit numerous health-promoting properties, especially in the enhancement of the immune system, as an antimicrobial agent, and a treatment for heart diseases [19]. Oleuropein, a glycosylated secoiridoid, is one of the most abundant bioactive compounds present in olive leaves, together with other phenolic substances, flavonoids, chalcones, and triterpenes [17,18]. Recently, numerous studies have proposed the utilization of olive leaves or olive leaf extracts in different food applications such as in herbal teas, food supplements, natural preservatives, antimicrobials and antioxidants in the meat, baking, dairy, and vegetable industries [20,21,22].

Nowadays, food industries pay great attention to producing healthier products endowed with high nutritional value and therapeutic benefits. For this reason, several studies have been carried out with the specific goal of replacing some conventional ingredients in the preparation of mayonnaise [12]. For this reason, in the current research, a novel seed oil blend, soymilk, and olive leaf vinegar were used as alternative ingredients. 

In our previous study [23], olive leaf vinegar was produced and its functional potential was assayed in an oil/vinegar dressing-based formulation. In the current research, the feasibility of using this olive leaf vinegar in mayonnaise formulation was investigated. The study was conducted by evaluating other unconventional ingredients, such as a vegetable oil blend (soybean and high oleic sunflower seed oil) and soymilk as an egg substitute. Chemical characterization of the formulated mayonnaise was performed through moisture, fat, pH, total phenols, and fatty acids profile determination. In storage, the oxidative stability of mayonnaise was evaluated by PV and oleuropein content determination. Finally, the sensory profile of mayonnaise was also developed. 

## 2. Materials and Methods

### 2.1. Materials

All the chemicals and solvents were of analytical or HPLC grade and supplied by C. Erba (Rodano, Milan, Italy), while the gallic acid, ascorbic acid, hydroxytyrosol, and oleuropein standards were purchased from Sigma Aldrich Chemicals Co. (Milan, Italy).

A vegetable oil blend, soymilk, two different kinds of vinegar, and salt were chosen as ingredients for mayonnaise. The soybean and high oleic sunflower seed oil, soymilk, and salt were purchased from a local supermarket, while the 18% acetic acid vinegar (AV) was kindly provided by A. Milano vinegar factory SRL, Acerra, Napoli. The oil blend (OB) and the olive leaf vinegar (OLV) were produced in our laboratory. OB was obtained by carefully mixing the soybean and sunflower oils in a ratio of 15:85 (*v*/*v*); OLV was obtained according to the procedure reported by De Leonardis et al. [23] with modifications. In brief, olive leaves were randomly harvested from local trees; thus, the leaves were layered up and left to dry spontaneously at room temperature and darkness for fifteen days; then, the dried leaves, in a ratio of 1:10 *w*/*v*, were homogenized with alcoholic vinegar (AV) in a blender and left to macerate for five days at room temperature. Finally, OLV was filtered through paper and stored at room temperature in darkness.

### 2.2. Preparation and Storage of Mayonnaise

The OB, soymilk, vinegars (AV or OLV), and salt were mixed in the following percentage: 56, 35, 8, and 1%, respectively. All the ingredients were homogenized at 20 °C with an immersion knife blender until a stable emulsion were formed. Then, the mayonnaise, after analysis (time zero), was packed in 50g-capacity closed glass jars and stored in different time and temperature conditions, such as (i) heat storage (H-S) in a thermostat at 40 °C for 2 months; (ii) room temperature storage (RT-S) in the dark at 18–24 °C for 12 months; (iii) cold storage (C-S) in a refrigerator at 4 °C for 12 months.

### 2.3. Chemical Determination

Moisture of the samples was determined by drying the mayonnaise sample in oven at 105 °C until constant weight; total lipid amount parameter was obtained by performing four repeated extractions with hexane in a ratio of 2:1 *v*/*v*. The mayonnaise samples were split into the aqueous and fat phases by the following method: 10 g mayonnaise and 2 mL methanol were vortexed for 3 min; after centrifugation (4000 rpm × 20 min), each layer was collected separately with a Pasteur pipette. Free acidity (% oleic acid) and peroxide value (PV, meqO_2_/kg) were determined according to the AOAC methods [24]. Fatty acid methyl esters were determined using a gas-chromatograph TRACE 1300 (Thermo Fisher Scientific SpA, Rodano, MI, Italy) equipped with a flame ionization detector and an Alltech EC-1000 FFAP (Alltech, Lexington, KY, USA) capillary column (30 m × 0.32 mm ID; film, 0.25 μm) at the following conditions: carrier gas He at 50 kPa; split injection system; splitting ratio of 1:50; injector and detector temperatures set at 250 °C and 270 °C, respectively; programmed ramp from 150 to 240 °C at 10 °C min^−1^; injected quantity of 1 μL; cold transesterification with 2 N methanolic potash.

Total phenols (TP) were determined on both the vinegars and mayonnaise aqueous layers employing the Folin–Ciocalteu reagent and UV–visible spectrophotometer Evolution TM201/220 (Thermo Fischer Scientific SpA, Rodano, MI, Italy); TP were quantified by means of a gallic acid standard calibration curve (gallic acid equivalent, GAE). HPLC phenolic analyses were carried out by an HPLC instrument model ProStar 230 pump and 330 PDA detector (Varian Analytical Instruments, Palo Alto, CA, USA) equipped with a Kinetex 5u C18 100 Å column (150 × 4.6 mm) (Phenomenex, Torrance, CA, USA). The mobile phase was a mixture of 0.2% *v*/*v* H_3_PO_4_ (eluent A), methanol (eluent B), and acetonitrile (eluent C); the gradient for the A/B/C eluents was as follows: 0 min, 96/2/2%; 24 min, 50/25/25%; 27 min, 40/30/30%; 36 min, 0/50/50%; 49 min, 96/2/2%; chromatograms were obtained at 280 nm; finally, independent hydroxytyrosol or oleuropein standard calibration curves (linear in the range of 0.05–0.50 mg/mL) were used for quantitative analysis. 

### 2.4. Antioxidant Activity by the DPPH Method

Variable amounts of methanol (DPPH blank solution) or mayonnaise aqueous layer were alternatively mixed with methanol (solvent) in plastic cuvettes to make the final volume of 2.5 mL; subsequently, the reaction solution was completed by adding 0.5 mL of DPPH (0.5 mM in absolute methanol). After 40 min incubation at room temperature, in darkness, the absorbance at 517 nm was read. The inhibition percentage was calculated using the following equation: %I = ((Abs blank − Abs sample)/Abs blank) × 100. IC_50_ (concentration ensuring 50% inhibition) was calculated by using the linear regression of %I vs. mg of mayonnaise sample.

### 2.5. Sensory Evaluation

Sensory evaluation was carried out after the first week of production. According to Pradhananga et al. (2015) [25], mayonnaise was served with bread to twelve trained panelists for an evaluation through a 9-point hedonic scale (9 = extremely like, 1 = extremely dislike). The median of the judges’ scores given to colour, odour, texture, taste and overall acceptability was calculated. 

### 2.6. Statistical Analyses

All the analyses were carried out in triplicate by expressing data as the mean ± standard deviation. Differences among the means were evaluated using Tukey’s HSD test at *p* ≤ 0.05 (ANOVA) with SPSS software version 26.0 (IBM Corp., Armonk, NY, USA).

## 3. Results and Discussion

### 3.1. Main Characteristics of Mayonnaise Ingredients

Analytical determinations carried out on the the mayonnaise ingredients is displayed in Table 1.

Commonly, low-cost vegetable seed oils, such as soybean, sunflower, rapeseed, and corn are mostly used in the formulation of conventional mayonnaise [15]. Oil plays a fundamental role in the rheological properties of mayonnaise, especially mouthfeel and texture. Moreover, oil also influences flavour, creaminess, palatability, appearance, and finally, shelf life which is affected mainly by the oxidation of unsaturated acids [12]. According to our previous work [23], the oil blend (OB) was obtained by merging high oleic sunflower seed oil (85%) with soybean oil (15%). High oleic sunflower oil was introduced in the 1970s when hybrid seeds were selected to reduce the oil’s linoleic acid content in favour of oleic acid [26]. From the nutritional and functional points of view, oleic acid evidenced a modulatory effect on health and prevention of cancer, autoimmune and inflammatory diseases [27]. Today, high oleic sunflower oil is used widely as a salad or cooking oil and in margarine and spread manufacturing. Nevertheless, high oleic sunflower oil is characterized by an irrelevant amount of linolenic acid (less than 0.1%) which is very high in soybean seed oil (up to 5%). Consequently, the OB was designed just to obtain an oil with a high content of both oleic acid and ω-3 essential unsaturated fatty acids. Indeed, the OB fatty acid profile was characterized by a content of 4.6% palmitic (C16:0), 74.6% oleic (C18:1n9), 14.7% linoleic (C18:2n6), and 0.9% linoleic (C18:3n3) acid, respectively (Table 1). The free acidity (0.2% oleic acid) and peroxide value (3.5 meqO_2_/kg) of the OB were very low and consistent with the refining processing conducted in the production of basic oils.

Soymilk was introduced as a replacement of egg yolk to obtain low-cholesterol vegetable mayonnaise with the same rheological and textural properties as the conventional ones. Furthermore, that choice could also satisfy vegan consumers. As reported in the literature [28], soymilk is rich in minerals (calcium, iron, magnesium, zinc, copper, and phosphorus) and vitamins (A, E, K, B1, B3, B5, B6, and folate). Furthermore, soymilk has a lower content of sodium, lactose, saturated fat, and cholesterol than cow’s milk. Reading its commercial label, soymilk is composed of water (89.7%), proteins (3.7%), carbohydrates (3.2%), sugar (2.8%), fat (2.3%), fibre (1.0%), and salt (0.1), respectively. Finally, the soymilk fatty acid profile (Table 1) was characterized by a content of 12.3% palmitic (C16:0), 43.7% oleic (C18:1n9), 32.1% linoleic (C18:2n6), and 5.0% linonelic (C18:3n3) acid, respectively.

Lemon, citric acid juices, or vinegar are commonly used in conventional mayonnaise as acidifying agents; these are added specially to adjust pH which plays a key role in the emulsion structure [1]. Moreover, acidifying agents also influence the taste (sourness) and smell of the final product. Since ancient times, several types of vinegar have been utilized in food preparation, as a functional drink or food ingredient, and for medical purposes [29]. A lot of studies have highlighted healthy vinegar properties, such as antibacterial and antioxidant activity; lipid metabolism regulation; weight loss; anticancer and antidiabetic effects; lowering cholesterol levels in the blood [29,30]. Recently, novel types of vinegar have been investigated, such as those obtained from onion juice, rubberwood, tomato, strawberry, pineapple, sweet potato, olive oil mill wastewaters, and others [31]. Olive leaf vinegar (OLV) represents a kind of revolution in the field of vinegar manufacturing [23]. The OLV used in this research was characterized by a 2.16 pH value and a high content of total phenols (7.2 mg/mL GAE) and oleuropein (6.0 mg/mL OLE), respectively (Table 1).

### 3.2. Main Characteristics of Formulated Mayonnaise

The main characteristics of the mayonnaise prepared with the oil blend, soymilk, and one of the two tested vinegars, OLV or AV, are reported in (Table 2).

No statistically significant difference were found between the mayonnaise samples (AV-May and OLV-May) with regard to pH, water, fat and fatty acids (Table 2). The values of pH were exceptionally low (2.15 on average) for both mayonnaises; the positioning in the acidity range was considered beneficial for the prevention of microbial spoilage [25]. Furthermore, pH affects deeply the emulsion structure of mayonnaise influencing the net charge of protein [14]. 

It should be recalled that soymilk was used as an egg replacement in the formulated mayonnaise. In the conventional mayonnaise, egg yolk is incorporated for its brilliant gelling, whipping, and emulsification properties [14]. The high emulsifying performance of egg yolk is due to the high content of lecithin; conversely, the possibilities of *Salmonella* spp. contamination, high cholesterol content and price still exist when using egg yolk [32]. Thus, the identification of egg replacements is a current research topic. Vegetable protein isolates, such as soy, pea, sunflower, tomato seeds, wheat, faba beans, and lupin, has been extensively tested as food emulsion stabilizers [33,34]. Soymilk used in cream-like emulsions with sunflower oil enjoyed encouraging findings in terms of texture stability and nutritional composition [35]. In the current study, despite the low pH value, direct visual observation of mayonnaise evidenced the absence of flocculation by confirming the stability of emulsion between the OB and soymilk. 

Chronic diseases, i.e. cardiovascular diseases and obesity, are correlated with an excessive intake of fat. Generally, fat content is more than 65% in conventional mayonnaise, which, for this reason, is considered a high-fat and high-calorie food. A low-fat emulsion is possible by decreasing the dispersed phase and increasing simultaneously the liquid phase. Park et al. (2020) [36] studied different starch pastes as a fat replacement in mayonnaise, modified by citric acid hydrolysis, annealing, octenyl succinic anhydride, acetylation, and heat moisture treatment. Here, the formulated mayonnaises were characterized on average by 57% and 35% content of total fat and moisture, respectively. Usually, the moisture content tends to increase with the addition of a fat replacement, especially with carbohydrate-based fat [37]. The found moisture value was lower than that prepared by Palma et al. (2004) [38] (42.0–47.0%) and higher than that of one processed with corn oil by Muhammad et al. (2013) [39] (15.2–15.3%). However, if fat content reduction is the main goal, at the same time, quality of the chosen fat is equally significant [40]. The formulated mayonnaise samples showed similar fatty acid profiles characterized on average by about 11, 64, and 23 g/100 g of saturated, monounsaturated, and polyunsaturated fatty acids, respectively (Table 2). The linolenic acid content (1.7% on average) was higher than that of the OB (0.9%, Table 1) thanks to the soymilk fat contribution. The increased value of this fatty acid improved the functional potential of mayonnaise due to the ω-3 fatty acids are strongly associated with a minor risk of cardiovascular diseases, control of long-term glycaemic index and insulin resistance [41]. In this regard, it is known that the ideal ω-6/ω-3 ratio should be 4:1, while it was calculated to be about 13:1 in the proposed mayonnaise. 

Moreover, the PV of mayonnaise was unchanged as compared to that of the OB; specifically, the PV was 2.0 and 1.9 meqO_2_/Kg in the freshly prepared AV-May and OLV-May, respectively. According to the recent work of Jadhav et al. (2022) [42], homogenization treatment and other ingredients did not trigger any auto-oxidation reactions in the formulated mayonnaises.

Furthermore, a significant difference emerged between AV-May and OLV-May regarding phenolic content and antioxidant activity (Table 2). A lot of natural vegetable phenolic compounds exhibit a high antioxidant activity due to their redox property. For example, olive oil phenols taken along with food can protect the human body against oxidative stress [43]. In the formulated mayonnaise, we expected that the antioxidant activity would be directly influenced by OLV addition and its phenolic content. 

Moreover, olive leaf phenols could contribute to an emulsion formulation capable of retaining its chemical, physical, and sensorial properties over time. Indeed, olive leaf phenols, thanks to their amphiphilic structure, could exert surface activity and influence the emulsification process [23]. As evidenced by the study of Giancitucci et al. (2016) [44], oleuropein and its derivatives significantly affect the dispersion degree of mayonnaise-like emulsions, influencing their microstructure, flow behaviour, and physical stability. 

Although the Folin–Ciocalteu method is less selective than HPLC analysis, it is a recognized and widely used technique for quantifying total phenolic compounds in various vegetable products. As recorded in Table 2, the total phenols of OLV-May were 0.68 mg/g GAE and only 0.13 mg/g GAE in AV-May. The oleuropein (OLE) content of OLV-May was 0.52 mg/g OLE, confirming that this compound was one of the prevailing compounds. Conversely, hydroxytyrosol was detected only in a trace amount (data not shown). A significant free radical scavenging activity was found in OLV-May, certainly correlated with the presence of olive leaf phenol compounds. Specifically, a DPPH IC_50_ of 0.10 mg/g was determined in OLV-May. This could be considered a very positive nutritional feature for our sauce helpful in overcoming some health concerns related to consuming conventional mayonnaise.

Summarizing the obtained results, the formulated OLV-May could be considered as a possible health mayonnaise from several points of view. In fact, taking into consideration the European Regulations [45,46], OLV-May could be considered a suitable source of the following compounds: (i) ω-3 fatty acids (because linoleic acid content was up to 0.3%); (ii) monounsaturated fat (because it was up to 45% on fatty acid composition and covered 20% of product energy); (iii) unsaturated fat (because it was up to 45% on fatty acid composition and covered 20% of product energy). Finally, it must be emphasized that the total phenols (68 mg per 100 g of product) and oleuropein (52 mg/100 g) amount found in OLV-May could contribute to the protection of blood lipids from oxidative stress, in line with what is admitted for olive oil polyphenols [45].

### 3.3. Mayonnaise Sensory Analysis

**Figure 1:**
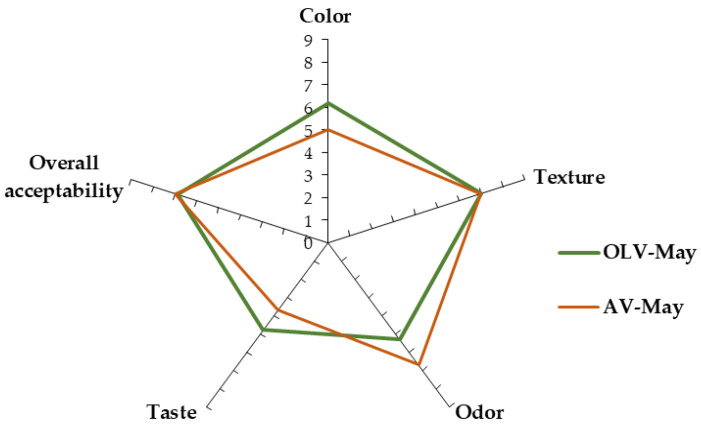
Spider plot median score (MS) of the 9-point hedonic scale of the sensory analysis of the mayonnaise samples prepared with olive leaf vinegar (OLV-May) and alcoholic vinegar (AV-May).

Certainly, sensory acceptance is one of the most important factors for the success of a ready-to-eat product, such as mayonnaise. Figure 1 shows the median score (MS) obtained for the colour, texture, odour, taste, and overall acceptability of the OLV-May and AV-May mayonnaises. 

From the analysis of the graph, it emerged that the sensory characteristics of AV-May and OLV-May were very close to each other. Colour and texture are the immediate parameters evaluated in food emulsions. AV-May showed a white colour, very different from the typical pale yellow colour of conventional mayonnaise associated with the presence of eggs; indeed, the AV-May’s MS for colour was only 5.0 (neither like nor dislike). Instead, OLV-May showed a pleasant light brown colour that was evaluated at 6.2 MS (slightly like) by the panelists. Conversely, no significant difference emerged regarding the texture evaluated visually by the panelists. In both samples, the sauces’ texture appeared homogeneous and well-structured, without evident phase separation; thus, the evaluation of texture was 7.0 MS (moderately like) for both mayonnaises. Therefore, in the current research, no influence of OLV phenols was observed on the sample texture, while De Bruno et al. (2021) [47] found a negative effect of phenol extracts on mayonnaise consistency.

The odour of the mayonnaise samples was evaluated as the combined perception of orthonasal and retronasal sensations. The MS attributed to AV-May was 5.3 due to the pungent, slightly disliked smell of vinegar, while the odor of OLV-May was greatly appreciated (6.7 MS) due to perceptible olive leaf sensation. 

High acidity (pH around 2.15) influenced the taste evaluation that was calculated to be 4.8 and 3.7 MS for AV-May and OLV-May, respectively. Presence of oleuropein in OLV-May resulted in bitterness perception as evidenced by the panelists, although the other ingredients (OB and soymilk) helped to mask the bitter taste. 

Finally, despite the data reported above displaying slight differences, the overall acceptability was evaluated by the panelists around 7 MS (moderately acceptable) for both mayonnaise samples.

### 3.4. Mayonnaise Oxidative Stability

**Figure 2:**
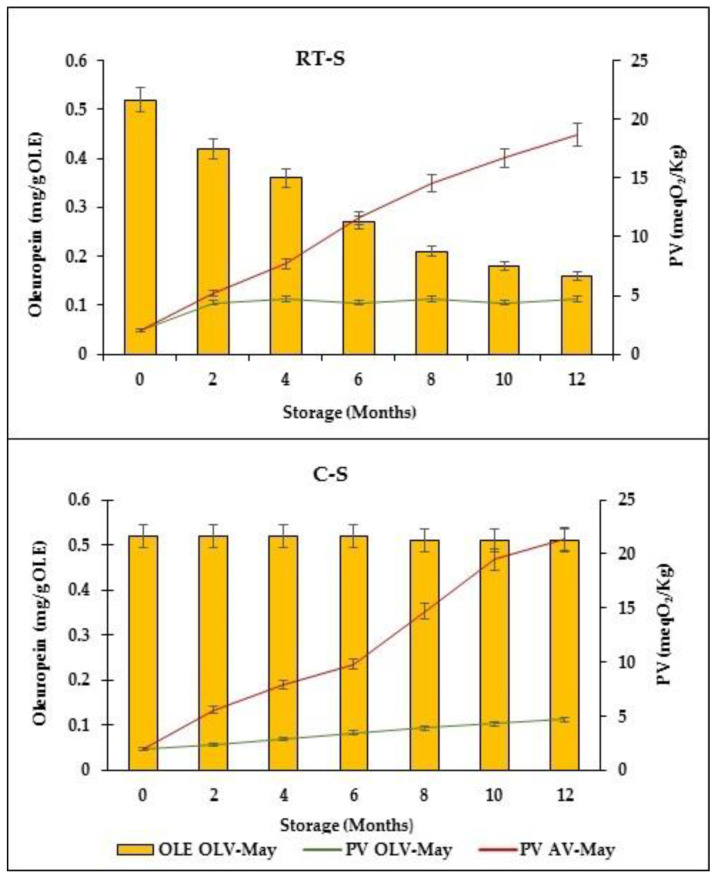
Variation of the oleuropein (OLE) content and peroxide value (PV) measured on the mayonnaise samples prepared with olive leaf vinegar (OLV-May) and alcoholic vinegar (AV-May) and stored at room temperature (RT-S) and cold storage (CT-S) for 12 months. The data are the means ± standard deviation of triplicate measures.

The shelf life of mayonnaise is strongly affected by autoxidation of the lipid fraction that causes an increase in the PV and the development of an unpleasant rancid smell [37,48]. Free radicals are the initial products formed because of this reaction; thus, measurement of the PV can help to estimate the oxidation stage [49]. Commonly, lipid exposure to air and light influences the PV; in addition, the surface of oil-in-water emulsion droplets may accelerate tremendously the lipid oxidation rate in mayonnaise [48,49]. In the current study, the mayonnaise samples were stored in closed glass jars in darkness in order to control air and light effects. PV and oleuropein changes determined in the samples stored at room (RT-S) and cold (CT-S) temperatures for up to 12 months is shown in Figure 2.

The oxidative stability of OLV-May was clearly higher than that of AV-May. Indeed, at the end of the storage period (12 months), the PV in OLV-May was found to be very low, specifically, 4.4 and 4.7 meqO_2_/kg under room and cold (4 °C) storage temperatures, respectively. Conversely, during the same period, the PV in the control mayonnaise (AV-May) was 18.5 and 21.4 meqO_2_/kg at room and cold temperatures, respectively. This was the evidence that the OLV phenols effectively exerted antioxidant activity in the mayonnaise as hoped in according with the literature [23,47,48,49,50,51]. Finally, significant and constant oleuropein reduction occurred in the OLV-May stored at room temperature (up to 70% after 12 storage months), while it remained unchanged in the sample stored under cold temperature.

**Figure 3:**
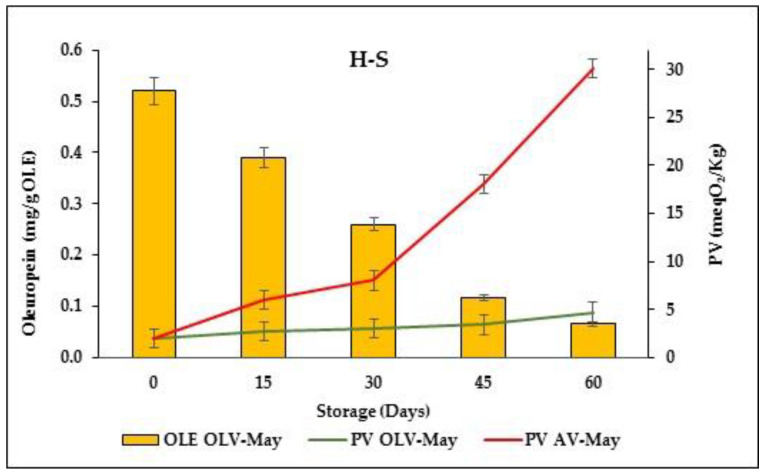
Variation of the oleuropein (OLE) content and peroxide value (PV) measured at 40 °C for 2 months in the mayonnaise samples prepared with olive leaf vinegar (OLV-May) and alcoholic vinegar (AV-May). The data are the means ± standard deviation of triplicate measures.

An accelerated oxidation test carried out in a thermostat at 40 °C for a maximum of two months confirmed what was observed in the above-discussed storage test (Figure 3). OLV-May showed a higher oxidation stability than AV-May, in which the PV increased from 2.0 to 30.1 meqO_2_/kg in 60 days, while very little change was observed for OLV-May (from 1.9 to 4.7 meqO_2_/kg). Moreover, as observed at room temperature (RT-S test), oleuropein decreased from 0.52 to 0.07 mg/g in the sample stored at 40 °C. 

It should be noted that at both storage conditions, depletion of oleuropein did not merge with the formation of hydroxytyrosol that was found only in trace (data not shown).

Therefore, this research demonstrated that olive leaf vinegar exerts a high antioxidant activity, confirming what was observed in our previous research [23] in which OLV increased the OB’s oxidative resistance by about 40% and 65% at elevated temperatures (Rancimat test at 130 °C) and at 40 °C (oven test), respectively. Moreover, in the same study [23], in an oven test, oleuropein depletion was observed without simultaneous formation of hydroxytyrosol. Therefore, the reduction of oleuropein during storage appeared to be temperature-dependent and caused by oxidative, rather than hydrolytic, phenomena. Nevertheless, this evidence was opposite to what was observed by De Leonardis et al. (2021) [52], namely that hydrolysis of oleuropein-like compounds occurred during long storage of extra virgin olive oils leaving hydroxytyrosol formation. However, what was observed here was connected with a reasonable assumption that in OLV, there are no active hydrolytic enzymes capable of degrading oleuropein. Nevertheless, oxidation of OLV phenols and its effects on the overall quality of formulated mayonnaise could be an interesting subject for further investigation.

## 4. Conclusions

In the current study, mayonnaise was fabricated by using alternative ingredients to meet increasing consumer requests for healthy food products. Mayonnaise was characterized and the effects of ingredients on its sensory and oxidative stability under different time/temperature conditions were evaluated.

The oil blend, obtained by merging soybean (15%) and high oleic sunflower (85%) seed oils, was demonstrated to be a well-designed option; in fact, for this typology of mayonnaise was characterized byan optimal content of monounsaturated and ω-3 fatty acids was found.

Furthermore, the choice of soy milk as an egg substitute proved to be a winning choice because a well-structured and stable emulsion as well a lower fat product were obtained. Moreover, soymilk may meet the needs of both health-conscious and vegan consumers that do not prefer animal-based products. 

However, olive leaf vinegar certainly represents the major novelty of the formulated mayonnaise. The presence of this particular vinegar typology ensures that the proposed mayonnaise could meet both nutritional and sustainability requirements. From the nutritional point of view, olive leaf vinegar supplemented the mayonnaise with bioactive and antioxidant compounds, especially oleuropein, which also improved positively the shelf life of the product. From the sustainability point of view, olive leaf vinegar can enhance the olive oil industry by-product valorization, implementing practices for a circular and sustainable economy.

Finally, from the consumer acceptability point of view, further research is needed to improve the sensory characteristics of the proposed mayonnaise.

## Figures and Tables

**Table 1:** Determined analytical characteristics of the mayonnaise ingredients. The data are the means ± standard deviation of triplicate measures.

Determinations	Oil Blend (OB)	Soymilk	Olive Leaf Vinegar (OLV)	Alcoholic Vinegar (AV)
pH			2.16 ± 0.06	2.15 ± 0.08
Total phenols (mg/mL GAE)			7.2 ± 0.8	–
Oleuropein (mg/mL OLE)			6.0 ± 0.5	–
Free acidity (% oleic acid)	0.2 ± 0.0			
Peroxide value (meq O_2_/kg)	3.5 ± 0.1			
Fatty acids (%)				
C14:0	0.15 ± 0.0	0.6 ± 0.0		
C16:0	4.6 ± 0.2	12.3 ± 0.6		
C16:1	0.1 ± 0.0	0.9 ± 0.0		
C17:0	0.1 ± 0.0	0.1 ± 0.0		
C17:1	trace	0.1 ± 0.2		
C18:0	3.1 ± 0.1	4.0 ± 2.2		
C18:1	74.6 ± 3.7	43.7 ± 1.6		
C18:2	14.7 ± 0.73	32.1 ± 0.3		
C18:3	0.9 ± 0.0	5.0 ± 0.1		
C20:0	0.3 ± 0.0	0.6 ± 0.1		
C20:1	0.3 ± 0.0	0.6 ± 0.1		
C22:0	0.9 ± 0.0	–		
C24:0	0.3 ± 0.0	–		

**Table 2:** Analytical determinations of the mayonnaise samples (AV-May: 18% alcohol vinegar mayonnaise sample; OLV-May: olive leaf vinegar mayonnaise sample) *.

Determinations	AV-May	OLV-May
Water (%)	31.6 ± 1.9 ^a^	31.0 ± 1.7 ^a^
Fat (%)	58.2 ± 2.6 ^a^	57.0 ± 2.4 ^a^
Saturated fatty acids (%)	11.4 ± 0.5 ^a^	11.1 ± 0.6 ^a^
Monounsaturated fatty acids (%)	21.8 ± 1.1 ^a^	21.3 ± 1.0 ^a^
Polyunsaturated fatty acids (%)	23.5 ± 1.0 ^a^	23.0 ± 0.9 ^a^
Linolenic acid (ω-3) (%)	1.7 ± 0.1 ^a^	1.7 ± 0.1 ^a^
pH	2.16 ± 0.45 ^a^	2.15 ± 0.38 ^a^
Total phenols (mg/g GAE)	0.13 ± 0.1 ^a^	0.68 ± 0.1 ^b^
Oleuropein (mg/g OLE)	–	0.52 ± 0.1
Peroxide value (meqO_2_/kg)	2.0 ± 0.1 ^a^	1.9 ± 0.1 ^a^
Antioxidant activity (IC_50_ DPPH, mg/g)	–	0.10 ± 0.0

* The data are the means ± standard deviation of triplicate measures. The means followed by a different letter are significantly different at an alpha level of 0.05 according to Tukey’s HSD test.

## Data Availability

The data are contained within the article.

## Abbreviations

AV	18% alcoholic vinegar
AV-May	Alcoholic vinegar mayonnaise
C-S	Cold storage
H-S	Heat storage
MS	Median score
OB	Oil blend
OLE	Oleuropein
OLV	Olive leaf vinegar
OLV-May	Olive leaf vinegar mayonnaise
PV	Peroxide value
RT-S	Room temperature storage
TP	Total phenols
